# Iodine Status and Iodised Salt Consumption in Portuguese School-Aged Children: The Iogeneration Study

**DOI:** 10.3390/nu9050458

**Published:** 2017-05-05

**Authors:** João Costa Leite, Elisa Keating, Diogo Pestana, Virgínia Cruz Fernandes, Maria Luz Maia, Sónia Norberto, Edgar Pinto, André Moreira-Rosário, Diana Sintra, Bárbara Moreira, Ana Costa, Sofia Silva, Vera Costa, Inês Martins, Francisca Castro Mendes, Pedro Queirós, Bruno Peixoto, José Carlos Caldas, António Guerra, Manuel Fontoura, Sandra Leal, Roxana Moreira, Irene Palmares Carvalho, Rui Matias Lima, Catia Martins, Cristina Delerue-Matos, Agostinho Almeida, Luís Azevedo, Conceição Calhau

**Affiliations:** 1 Center for Health Technology and Services Research (CINTESIS), 4200-450 Porto, Portugal; mariadaluz.maia@gmail.com (M.L.M.); sonia.norberto.nutri@gmail.com (S.N.); andrerosario79@gmail.com (A.M.-R.); diana-sintra@hotmail.com (D.S.); barbaramoreirapsicologa@gmail.com (B.M.); ana.fontescosta@gmail.com (A.C.); sofiasilva78@hotmail.com (S.S.); verarcosta@gmail.com (V.C.); minesfmartins@gmail.com (I.M.); francisca_castromendes@hotmail.com (F.C.M.); pdqueiros@gmail.com (P.Q.); bruno.peixoto@iucs.cespu.pt (B.P.); carlos.caldas@iucs.cespu.pt (J.C.C.); ajmonicaguerra@hotmail.com (A.G.); manfontoura@gmail.com (M.F.); leal.sc@gmail.com (S.L.); irenec@med.up.pt (I.P.C.); lfazevedo55@gmail.com (L.A.); ccalhau@nms.unl.pt (C.C.); 2 Department of Biomedicine–Biochemistry Unit, Faculty of Medicine of the University of Porto, 4200-450 Porto, Portugal; 3 Nutrition & Metabolism, NOVA Medical School|Faculdade de Ciências Médicas, Universidade Nova de Lisboa, 1169-056 Lisbon, Portugal; 4 LAQV/REQUIMTE–Instituto Superior de Engenharia, Instituto Politécnico do Porto, 4249-015 Porto, Portugal; cmm@isep.ipp.pt; 5 LAQV/REQUIMTE-Department of Chemical Sciences, Faculty of Pharmacy, University of Porto, 4249-015 Porto, Portugal; edgarpinto7@gmail.com (E.P.); almeida@ff.up.pt (A.A.); 6 Department of Community Medicine, Information and Health Decision Sciences (MEDCIDS), Faculty of Medicine, University of Porto, 4200-450 Porto, Portugal; 7 CESPU, Institute of Research and Advanced Training in Health Sciences and Technologies, 4585-116 Gandra, Portugal; roxana.moreira@iucs.cespu.pt; 8 Division of Paediatric Nutrition, Department of Paediatrics, Integrated Paediatric Hospital, Centro Hospitalar São João, Porto. Faculty of Medicine, University of Porto, 4200-450 Porto, Portugal; 9 Division of Paediatric Endocrinology, Department of Paediatrics, Integrated Paediatric Hospital, Centro Hospitalar São João, Porto. Faculty of Medicine, University of Porto, 4200-450 Porto, Portugal; 10 Department of Biomedicine-Anatomy Unit, Faculty of Medicine, University of Porto, 4200-450 Porto, Portugal; 11 Department of Clinical Neurosciences and Mental Health, Faculty of Medicine, University of Porto, 4200-450 Porto, Portugal; 12 Directorate-General of Education, 1049-005 Lisbon, Portugal; matias.lima@gmail.com; 13 Obesity Research Group, Department of Cancer Research and Molecular Medicine, Faculty of Medicine, Norwegian University of Science and Technology (NTNU), NO-7491 Trondheim, Norway; catia.martins@ntnu.no

**Keywords:** iodine status, children, urinary iodine, salt iodisation, salt intake, monitoring, public health

## Abstract

The World Health Organization promotes salt iodisation to control iodine deficiency. In Portugal, the use of iodised salt in school canteens has been mandatory since 2013. The present study aimed to evaluate iodine status in school-aged children (6–12 years) and to monitor the use of iodised salt in school canteens. A total of 2018 participants were randomly selected to participate in a cross-sectional survey in northern Portugal. Children’s urine and salt samples from households and school canteens were collected. A lifestyle questionnaire was completed by parents to assess children’s eating frequency of iodine food sources. Urinary iodine concentration (UIC) was measured by inductively coupled plasma-mass spectrometry. The median UIC was 129 µg/L which indicates the adequacy of iodine status and 32% of the children had UIC < 100 µg/L. No school canteen implemented the iodised salt policy and only 2% of the households were using iodised salt. Lower consumption of milk, but not fish, was associated with a higher risk of iodine deficiency. Estimation of sodium intake from spot urine samples could be an opportunity for adequate monitoring of population means. Implementation of iodine deficiency control policies should include a monitoring program aligned with the commitment of reducing the population salt intake.

## 1. Introduction

Iodine is a key micronutrient for the synthesis of thyroid hormones, which are essential for healthy growth, particularly for normal neurological development [1]. Chronic inadequate iodine consumption at an early age may have long term implications in a number of cognitive outcomes including reduced intelligence quotient [2,3,4]. 

Globally, the elimination of iodine deficiency among vulnerable populations including school-aged children and pregnant women is regarded as a major public health challenge. Accordingly, the World Health Organization (WHO) promotes salt iodisation at a global level as a cost-effective and safe strategy to control iodine deficiency.

Across Europe, strategies to control and monitor iodine deficiency are limited to a number of countries. Concerns related to the potential adverse consequences of iodine fortification, such as mandatory salt iodisation programs, may delay the implementation of those strategies. Nevertheless, the risks of iodine excess are generally considered to be low and are far outweighed by the substantial risks of iodine deficiency [5].

The implementation of iodine fortification policies is more efficient when integrated with monitoring programs. The importance of these programs is well evidenced by the reappearance of iodine deficiency in the UK, a country that was known as iodine sufficient in the 1990s that had no iodine control or monitoring programs [6].

In Portugal, a national representative study conducted among pregnant women in 2010 indicated an iodine status well below the WHO adequacy interval recommended for this population (150–249 µg/L), reporting median urinary iodine concentration (UIC) values ranging from 50 µg/L in the region of Azores to 84.9 µg/L in continental Portugal [7]. Another survey reported in 2012 that Portuguese school-aged children had a median UIC of 106 µg/L [8], which is within the adequacy interval of 100–199 µg/L recommended by the WHO for this population group. Nevertheless, it was highlighted that 47% of this young population had a median UIC below 100 µg/L. 

Currently, household and industry usage of iodised salt is voluntary and no regulation exists regarding the concentration of iodide/iodate in fortified salt. Interestingly, the use of iodised salt for meal preparation in school canteens has been mandatory since 2013, but no data exists regarding the adherence of schools to this policy, which emphasises the lack of a regulatory framework and surveillance in the country [9]. Accordingly, the present study aimed to evaluate iodine status in school-aged children and to monitor the use of iodised salt in school canteens and households.

## 2. Methods

### 2.1. Participants and Study Design

A cross-sectional survey was conducted in three regions of northern Portugal (Tâmega, Grande Porto, and Entre Douro e Vouga) to evaluate iodine status in a school-aged population (6–12 years). Tâmega is an inland region known for having in the past a great incidence of thyroid disease, socioeconomic disadvantages, inequalities, and urban-to-rural contrasts. Grande Porto is the most socio-economically developed region in the North of Portugal and it is a coastal region known to have urban-to-suburban-to-rural contrasts. The region of Entre Douro e Vouga is a smaller coastal region with some areas of socioeconomic disadvantage. Geographical differences between these regions provided an opportunity to investigate the influence of living close to the sea on iodine status. A multi-stage sampling method, with clusters at three levels (county, school clusters, and school classes), was implemented to select classes from elementary and middle schools (1st to 6th grade) to provide a representative sample of the population. The 83 schools and 32 school clusters were selected according to the number of children and with stratification for the number of clusters by county and for the type of schools (elementary versus middle). A formal sample size determination was undertaken to ensure the estimation of proportions with an expected margin of error of 3%, assuming a design effect between 1.5 and 2.0 (accounting for the multi-stage complex sampling method), and an intended confidence level of 95%. Based on these assumptions, a sample size higher than 2000 children was needed.

### 2.2. Data Collection

The study was approved by the Ethical Committee of S. João Hospital Center/Faculty of Medicine of the University of Porto. Data collection was approved by the National Committee for Data Protection and by the Directorate-General of Education. Recruitment and data collection was conducted between December 2015 and May 2016.

To improve the participation rate, selected school clusters were first invited by the Directorate-General of Education to take part in the survey. In addition, a first visit to the school clusters, involving teachers and school board directors, was promoted by the study researchers to schedule the field work activities, to ensure teachers’ collaboration in recruiting children, and to provide codified sealed kits to teachers of the selected classes. These kits included a consent form, lifestyle and behaviour questionnaires, and urine and salt collection containers. Upon informed consent, participants were asked to provide a first morning urine sample collected at home on the field work day, along with a sample of household salt. These samples, together with the questionnaires completed by parents, were returned to the researchers on the same day. A salt sample used for school meal preparation was also collected in the school canteens. The lifestyle questionnaire was specifically designed for this study and applied in a pilot study to check for inconsistencies, and assess the applicability and data entry protocols. Validation was not conducted as questions were treated independently and aimed to categorize groups. The questionnaire included questions regarding eating frequency (i.e. never, less than once a month, less than once a week, 2–3 times a week, once a day, or more than twice a day) of iodine natural sources (seafood) and iodine fortified food sources (premade baby cereal). Information on iodised salt consumption and awareness, socio-economic and educational background, and living conditions were also collected. Data regarding the consumption of milk products and eggs by children were collected from an additional online questionnaire completed by a sub-sample of parents (*n* = 615) as these questions were not included in the lifestyle questionnaire.

Anthropometry parameters including height (Seca^®^, model 213), weight (Tanita^®^, model UM-076), and waist circumference were measured in duplicate by trained researchers following published guidelines [10]. A cognitive performance assessment was also conducted on the field work day, but the results are out of the scope of the present paper. Children aged over 12 were excluded from data analysis for consistency with the age group of the WHO iodine nutrition guidelines.

### 2.3. Biochemical Analysis

#### 2.3.1. Iodine Determination in Urine Samples

Urinary iodine excretion was measured by inductively coupled plasma-mass spectrometry (ICP-MS), according to the method developed by the Centers for Disease Control and Prevention (CDC) [11].

ICP-MS analyses were performed using an iCAP™ Q instrument (Thermo Fisher Scientific, Bremen, Germany), equipped with a MicroMist™ nebulizer, a baffled cyclonic spray chamber (Peltier-cooled), a standard quartz torch, and a two-cone design (nickel sample and skimmer cones). High purity (99.9997%) argon (Gasin, Portugal) was used as a nebulizar and as a plasma gas source. The ICP-MS instrument operational parameters were as follow: RF power (1550 W); plasma gas flow (14 L/min); auxiliary gas flow (0.8 L/min); nebulizer flow rate (0.95 L/min). The equipment control and data acquisition were made through the Qtegra software (Thermo Fisher Scientific). The iodine (^127^I) isotope was monitored for analytical determination, and the tellurium (^125^Te) isotope was monitored as an internal standard. The instrument was tuned daily for maximum signal sensitivity and stability as well as for low oxides and doubly charged ion formation using the Tune B iCAP Q solution (Thermo Fisher Scientific; 1 μg/L of Ba, Bi, Ce, Co, In, Li, and U in 2% HNO_3_ + 0.5% HCl).

All the solutions were prepared with 1.0% (*v*/*v*) tetramethylammonium hydroxide, TMAH 25% *w*/*w* (Alfa Aesar, Karlsruhe, Germany), 0.01% Triton™ X-100 (Sigma-Aldrich, St. Louis, MO, USA), and 10 µg/L Te (Sigma-Aldrich, St. Louis, MO, USA). The calibration curve was obtained with six solutions of iodine concentrations within the 25–1000 µg/L range. The calibration standard solutions were prepared by adequate dilution of the iodine standard (Plasma CAL, SCP Science, Quebec H9X 4B6, Canada). The internal standard solution was added to all samples and to the standard solutions in order to obtain a 10 µg/L final concentration. Urine samples were diluted (10-fold) before analysis. The base urine used in this method was a pool of the urine samples from several participants. For analytical quality control purposes, certified reference materials, Trace Elements Urine, L1 and L2 (Seronorm™, Sero, Billingstad, Norway), were analyzed under the same conditions as the study participants’ samples.

#### 2.3.2. Creatinine, Sodium, and Potassium Determination

Creatinine, sodium, and potassium measurements were performed using an ADVIA 1800 instrument (Clinical Chemistry System, Siemens, Erlangen, Germany) by a certified laboratory. Urinary creatinine was determined by Jaffe’s reaction and sodium and potassium levels were determined using ion selective electrodes, following the manufacturer’s instructions.

Considering the importance of integrative approaches to monitor salt and iodine consumption for effective salt iodisation programs, the validated INTERSALT equations (a) and (b) to estimate 24-h urinary sodium excretion in young adults were applied [12,13]. This approach was evaluated by comparing our results with recent data from a 24-h urine excretion in a similar Portuguese population [14].

(a) For men: predicted 24-h urinary sodium excretion (mg/day) = 23 × {25.46 + [0.46 × spot sodium (mmol/L)] − [2.75 × spot creatinine (mmol/L)] − [0.13 × spot potassium (mmol/L)] + [4.10 × BMI (kg/m2)] + [0.26 × age (y)]}
(b) For women: predicted 24-h urinary sodium excretion (mg/day) = 23 × {5.07 + [0.34 × spot sodium (mmol/L)] − [2.16 × spot creatinine (mmol/L)] − [0.09 × spot potassium (mmol/L)] + [2.39 × BMI (kg/m2)] + [2.35 × age (y)] – [0.03 × age2 (y)]}

### 2.4. Iodine Determination in Salt Samples

The determination of iodine in salt samples was performed by ICP-MS. The methodology was optimized and validated, based on the CDC method [11]. For the analysis, salt samples were initially dissolved in ultrapure water (arium^®^ pro system, Sartorius, Göttingen, Germany) at a concentration of 10 g/L and then diluted 1:10. 

### 2.5. Statistical Data Analysis

Statistical analyses were carried out using SPSS 23 (IBM) [15]. Descriptive statistics are presented as numbers and percentages for categorical variables, as the mean and standard deviation (SD) for continuous variables or as the median and inter-quartile range (IQR–25th percentile–75th percentile) if the variable empirical distribution function was skewed. When testing hypotheses about continuous variables, parametric tests (Student’s *t* test and one factor analysis of variance-ANOVA) and nonparametric tests (Mann-Whitney and Kruskal-Wallis tests) were used as appropriate, taking into account normality assumptions and the number of groups compared. The Kolmogorov-Smirnov test was used to test normality assumptions of the variable distributions. When testing hypotheses about categorical variables, the Chi-square test and Fisher’s exact test were used as appropriate. 

The median UIC was used to compare iodine status between groups as recommended by the WHO. Parameter estimates and hypothesis tests were adequately adjusted and weighted taking into account the multi-stage complex sampling design used.

Univariate and multivariate weighted logistic regression models were used to assess factors associated with urinary iodine deficiency (concentration below < 100 µg/L). The dependent variable in all models was the urinary iodine deficiency. Independent variables were as indicated in the tables. The model goodness-of-fit was assessed by the Hosmer-Lemeshow statistic and test. The discriminative/predictive power of the model was evaluated by ROC-receiver operating characteristic-curve analysis. The influence of outlier data values on model fit was estimated using leverage statistics, and collinearity was assessed by evaluation of the coefficients correlation matrix. The results are presented as crude and adjusted Odds Ratios (OR) and their respective 95% confidence intervals.

The statistical significance level was set at 5% and differences were considered statistically significant when *p* < 0.05. 

## 3. Results

### 3.1. Sample Characteristics

**Figure 1:**
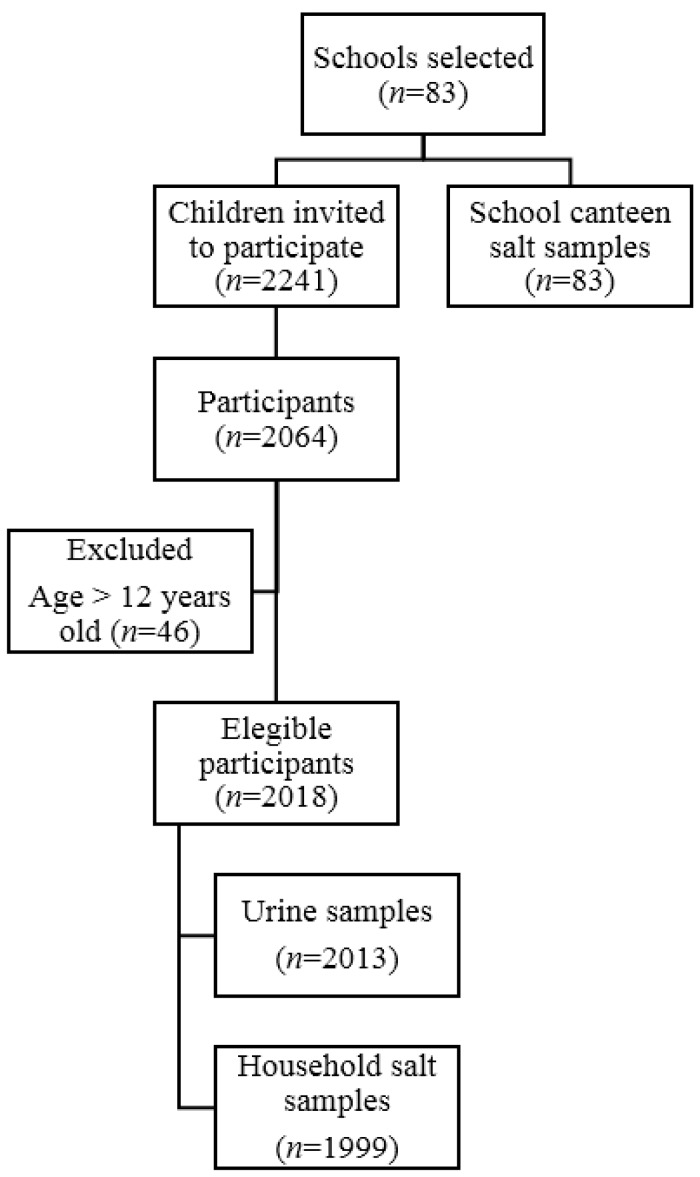
Recruitment diagram.

A total of 2018 schoolchildren aged 6–12 years old were recruited from 83 elementary and middle schools to participate in the survey (Figure 1). Urine and household salt samples were obtained from 2013 and 1999 participants, respectively. All invited schools took part in the study and provided a sample of the salt used for meal preparation in the canteens. The participation rate in the study was 92%.

The sample characteristics by regions are described in Table 1. The whole study sample included 52% (*n* = 1050) boys and 48% (*n* = 968) girls. No significant differences were found for sex and age distribution by regions. There were significant differences for body mass index (BMI) distribution by region (*p* = 0.021). Additionally, distribution by sex was similar in the different age groups (data not shown) (*p* = 0.354).

### 3.2. Urinary Iodine Concentration

#### 3.2.1. Iodine Status by Region

**Figure 2:**
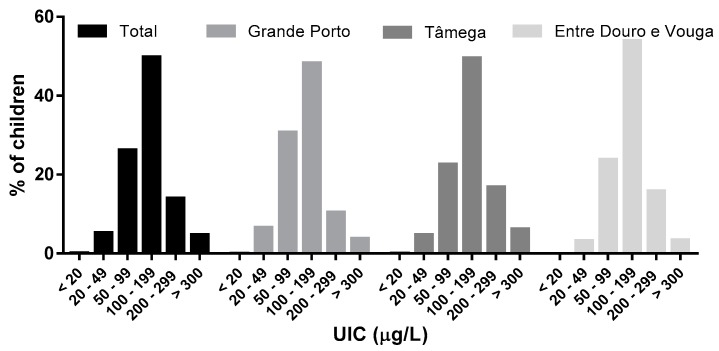
Urinary iodine distribution among schoolchildren according to the World Health Organization criteria for iodine adequacy.

The median UIC was 129 μg/L which is within the iodine adequacy interval of 100–199 μg/L recommended by the WHO (Table 1). The median iodine-to-creatinine ratio was 126 μg/g. Significant differences were found for the median UIC between regions, ranging from 116 μg/L in Grande Porto to 137 μg/L in Tâmega and 138 μg/L in Entre Douro e Vouga (*p* < 0.001). Additionally, the proximity to the coast had no impact on iodine status (data not shown). The proportion of the total population with UIC below 100 μg/L was 32%, ranging from 27% in Entre Douro e Vouga to 37% in Grande Porto (Figure 2). Additionally, outside the range of adequate iodine values, 5% of the population displayed elevated iodine levels. 

#### 3.2.2. Iodine Status by Sex and Age Group

The median UIC in boys was significantly higher than in girls (134 vs. 123 μg/L, respectively; *p* = 0.002) (Table 2). These differences remained significant when urinary iodine was adjusted for creatinine (Table 2; *p* = 0.002). Accordingly, the proportion of the population with UIC below 100 μg/L was significantly lower in boys than in girls (29% vs. 34%, respectively; *p* = 0.005). When examining UIC >300 μg/L, 6% of boys had excessive iodine levels compared to 3% of girls.

UIC also varied with age (Table 2). Specifically, the median UIC decreased significantly with age, ranging from 157 μg/L in the youngest group (5–6 years old) to 115 μg/L in the oldest group (11–12 years old) (*p* < 0.001). When adjusted for creatinine, this variation was even more marked with values ranging from 189 μg/g in the youngest to 89 μg/g in the oldest groups (*p* < 0.001).

The analysis of the proportions of the age groups according to the WHO cut-off for UIC adequacy categories are shown in Table 2. These data indicate that 20% of the children 5–6 years old were below 100 μg/L compared to 39% in the 11–12 years age group (*p* < 0.001). In contrast, 10% of the youngest age group had excessive iodine intake compared to 2% in the oldest age group (*p <* 0.001). 

#### 3.2.3. Iodine Status and Dietary Habits

Table 3 examines the associations of dietary habits with iodine status. Milk, yogurt, premade baby cereal, and household iodised salt, but not fish and egg consumption, were significantly associated with iodine status. Questionnaires completed by parents regarding iodised salt awareness and consumption indicated that 68% had never heard of iodised salt. In addition, only 8% of the families in the studied population reported using iodised salt at home. Comparison between self-reported iodised salt consumers and non-consumers initially showed no impact on iodine status. However, the ICP-MS analysis of iodine concentration in salt samples of the self-reported iodised salt consumers (8% of the population) confirmed that only 16% of this group was actually consuming iodised salt, corresponding to less than 2% of the whole population (*n* = 26). Iodised salt consumers had significantly higher UIC levels when compared to non-iodised salt consumers (*p* < 0.001) and iodine content in household iodised salt samples ranged from 16 mg/kg to 54 mg/kg (*n* = 26). Although all school canteens were providing lunch meals to the school community, none was using iodised salt (*n* = 83).

Examination of the impact of milk consumption on iodine levels showed that the group of children that consumed <1 glass of milk/day had a median UIC <100 μg/L (96 μg/L). This group corresponded to 23% of the population. In addition, 50% of the children that consumed <1 glass of milk/day and only 20% of the children that consumed at least two glasses of milk a day had a UIC <100 μg/L (*p* < 0.001). As expected, the risk of UIC <100 μg/L was significantly related with milk consumption (Table 4). Multivariate weighted regression models fully adjusted for age, sex, and other food items revealed that the OR of having a UIC <100 μg/L is 3.85 times higher with the consumption of <1 glass of milk per day compared to ≥2 glasses milk/day (OR = 3.85; 95% CI (2.42–6.13); *p* < 0.001). In addition, the OR of having a UIC <100 μg/L is 2.2 times higher with the consumption of 1 glass of milk/day compared to ≥2 glasses milk/day (OR = 2.20; 95% CI (1.45–3.34); *p* < 0.001). 

The risk of inadequate iodine consumption among this group (<1 glass of milk/day) was also significantly higher when compared to those consuming 1 glass of milk/day. The analysis of milk consumption also showed that older children consume less milk than younger children (*p* = 0.045) (Table 5), but age remained an iodine deficiency risk factor even when adjusted for milk consumption (Table 4). The population mean salt intake was 6.4 g/day, as estimated by sodium excretion in the spot urine samples (Table 1). The OR of having a UIC <100 μg/L is 2.2 times higher with the consumption of 1 serving of milk per day compared to two or more servings of milk per day (after adjusting for age and sex).

## 4. Discussion

The present study evaluated the iodine status of school-aged children in the north of Portugal. According to the WHO criteria for iodine status, the median UIC of 129 μg/L indicates an adequate status in this population group, which is in line with the previous national survey conducted in the country [8]. Our data were collected in the northern regions of Grande Porto, Tâmega, and Entre Douro e Vouga between December 2015 and May 2016. While in 2012 Grande Porto had a median UIC of 95 μg/L and 54% of the population below 100 μg/L [8], our study indicates a median UIC increase to 116 μg/L and a smaller proportion of the population in this particular region with UIC <100 μg/L (37%). Although the regions of Tâmega and Entre Douro e Vouga were not included in the previous published national survey [8], the prevalence of iodine deficiency in this population may have decreased in the recent years. In the previously published data [8] it was reported that 47% children had UIC values <100 μg/L, which contrasts with 32% in the present study.

In our study, proximity to the sea had no impact on iodine status. Actually, it was observed that the coastal region of Grande Porto had a higher prevalence of iodine deficiency when compared to the innerlands of Tâmega and Entre Douro e Vouga. In contrast, in the past, iodine deficiency and endemic goitre were more likely to be found in mountain and innerland areas in Portugal [16]. In the recent decades, a shift towards a reduction in iodine consumption disparities between these regions has been observed [8]. Interestingly, populations from the Azores and Madeira islands were shown by Limbert et al. to be at higher risk of iodine deficiency, confirming that proximity to the sea is not a major determinant of iodine status in Portugal [7]. Regarding iodine status by sex, we also found that boys had higher iodine status when compared to girls. This could be related to a higher energy intake by boys, as observed by Johner et al. [17]. 

Across Europe, there has been impressive progress in iodine deficiency control in the last few decades. However, sustaining this progress remains a great challenge as it requires close collaboration between partners at different levels and a strong partnership with the salt industry [18]. In a few countries where mandatory iodine fortification was implemented, an increased prevalence of excessive iodine intake among schoolchildren has been reported [19,20,21]. This is an important outcome to be monitored in order to re-evaluate iodine nutrition programs, for example in terms of the adjustment of the iodine concentration in fortified salt.

Excess iodine intake is related to an increased risk for hyperthyroidism and autoimmune thyroid diseases, though it is well-tolerated in most people [22]. In individuals with past or present thyroid abnormalities, even modest increases in iodine intake and excessive intakes can precipitate thyroid disorders [23]. In general, iodine intake should not exceed 500 μg/day, especially in countries with a history of iodine deficiency [18]. Nevertheless, it is recognized that the benefits of preventing poor cognitive outcomes from iodine deficiency far outweigh the side-effects of a mildly excessive iodine consumption [18]. While in Portugal this may not represent a problem, as only 5% of the studied population had excessive iodine intake, it is important to consider this group in future monitoring interventions in the country.

The percentage of parents who reported the household usage of iodised salt in our study was just 8% but among these, only 20% were actually using iodised salt, corresponding to less than 2% of the whole population. It was evident that many respondents were convinced that regular cooking salt is a natural source of iodine. These results further indicate that iodised salt consumption in the population is far from the WHO recommendation of 90% household coverage. Similarly, no school canteen (*n* = 83) was using iodised salt, which demonstrates the lack of efficacy and regulation regarding the current policy of iodised salt usage in Portuguese schools [9]. The fact that 92% of the participating children have lunch in school canteens at least once a week (information collected in the lifestyle questionnaire) suggests that this policy can be a potential effective iodine deficiency control intervention. 

The WHO claims that actions towards salt intake reduction and salt iodisation for the control of iodine deficiency disorders are compatible and feasible through the implementation of integrative programs. This relies on the fact that the concentration of iodine added to salt can be adjusted for salt intake [24]. This is a cost effective measure, beneficial for public health, particularly in countries with excessive sodium consumption like Portugal [25]. The opportunity to monitor both iodine and sodium excretion in a large sample lead us to estimate salt consumption in our study population. In spite of the limitation of applying the sodium excretion estimation equation validated for young adults, our results are comparable to those of a 24-h sodium excretion estimated among Portuguese children (6.4 ± 2.0 g/day in our study vs. 6.5 ± 2.2 g/day in Correia-Costa et al.) [14]. The estimation of sodium intake in high risk iodine deficiency population groups from spot urine samples could be an opportunity for adequate monitoring of population means and progress towards public health goals [26]. The fact that sodium intake in our population was estimated to be higher than that recommended by the WHO (<2 g/day) reinforces the importance of integrating salt iodisation and sodium intake reduction interventions in Portugal [25].

While previous Portuguese legislation on iodised salt recommended iodine fortification with potassium iodate at 25–35 mg/kg, which has been a guideline for the salt industry, at the moment no policy exists regarding the adequate concentration intervals for iodine fortification. Lack of cooperation and regulation in the salt industries has led to inadequate iodine prophylactic programs in several countries [19,21,27,28]. The broad iodine concentration range, between 16–54 mg/kg, observed in Portuguese household iodised salt clearly indicates a lack of control and a higher risk for the ineffective usage of iodised salt in the country. This may be a relevant aspect to consider in a future iodine deficiency control program to be implemented in Portugal.

Regardless of the lack of an effective iodine policy in Portugal, it could be assumed that iodine status has been improving among Portuguese schoolchildren suggesting a silent iodine prophylaxis. The fact that the household use of iodised salt is almost neglected suggests that other factors may be contributing to this improvement compared to the results found in the Limbert study [8]. Among these factors, population age, food consumption, study design, and analytical methodologies could explain these differences. It is our opinion that this comparison is extremely relevant for monitoring purposes. While both studies have included children aged 6–12 years, it was not possible to compare the mean age between them to explore potential differences. As age is a determinant of iodine status, this would be relevant, as food consumption and metabolism requirements during growth may change significantly. Milk consumption was confirmed to be a major determinant of iodine intake and was significantly lower in older compared to younger children. Bath and colleagues have also suggested that iodine status in teenage girls compared to younger girls may be explained by a higher milk consumption in the younger group [29]. Thus, it is not surprising that in our study the proportion of iodine deficient children is almost double in the oldest age group (11–12 years) compared to the youngest age group (5–6 years). We could argue that iodine status differences observed between the present study and Limbert’s study [8] could be due to different methodological approaches, such as urine sampling time or the iodine quantification method used. While total 24 h urine iodine excretion is considered the gold standard for iodine estimation, the use of alternative sampling methods are considered suitable, easier, and more practical to use in population studies [30]. Importantly, the WHO epidemiological criteria for assessing iodine nutrition in schoolchildren is based on the median UIC obtained from spot urine samples. Nevertheless, because urinary iodine excretion is affected by the circadian rhythm, single spot measures can fluctuate during the day [30]. The uniformity of urine sampling methods and analytical methods is desirable to improve the quality of monitoring studies. Regarding methodological approaches for iodine quantification, the use of ICP-MS in our study has offered a reliable, sensitive, and fast method to determine iodine concentration in the population. 

In our study, the importance of milk products consumption, but not fish, to the iodine adequacy in the population was evident. Despite the fish iodine content, the frequency of fish intake may not be sufficient to have an impact on iodine status in children. Limbert’s group also reported a reduced iodine deficiency risk among schools that provided milk to children supported by the national program for milk consumption in schools. However, no individual information was collected in that study. In our study, self-reported data from more than 600 households has confirmed that lower milk consumption increased the risk of iodine deficiency. One limitation of this approach was the fact that the data regarding consumption of milk products and eggs by children were not included in the initial lifestyle questionnaire. These data were collected in a second wave of data collection in an additional online questionnaire completed by a sub-sample of parents. Importantly, we identified that the group of children that consumes less than one glass of milk a day (23% of the population) had a median UIC <100 μg/L. The identification of this iodine deficiency risk group can be relevant for national public health entities. 

Considering that iodised salt usage is very limited in the country, milk products remain the major iodine source in the population. The importance of milk and dairy consumption to iodine prophylaxis among schoolchildren across Europe is clear. In countries like France, Germany, Spain, and the UK where iodised salt is not mandatory, milk consumption is a determinant of iodine optimal levels among schoolchildren [17,29,31,32,33]. However, when examining iodine nutrition data from children and pregnant women in the same countries across Europe, it seems evident that pregnant women are at a higher risk for iodine deficiency and may rely more on iodised salt consumption or supplementation to achieve adequate iodine levels compared to children. Thus, it is not surprising that in Portugal, where the usage of iodised salt is one of the lowest in Europe, higher iodine deficiency prevalence among pregnant women is also observed. Salt iodisation can be effective in preventing iodine deficiency in population groups that have a limited consumption of iodine rich foods such as milk products. In addition, pregnant women who are at a higher risk for iodine deficiency in Portugal could benefit from such intervention. Indeed, the WHO and the United Nations Children’s Fund (UNICEF) recommend iodine supplementation for pregnant and lactating women in countries where household iodised salt usage is less than 20% [34].

In conclusion, although the present study indicates that the average UIC in school-aged children is within adequacy levels, one third of the population may be at risk of iodine deficiency. While milk consumption is an important determinant of iodine status, the use of iodised salt in Portugal remains far from reaching the international guidelines. Whether or not iodine deficiency control policies are implemented in the country, we stress the need for a monitoring program and regulations aligned with the commitment of reducing population salt intake for an effective public health intervention. 

## Figures and Tables

**Table 1:** Population characteristics by region.

Variables	Total	Grande Porto	Tâmega	Entre Douro e Vouga	*p* Value
Whole (*n*)	2018	837	831	350	
Gender					0.153 ^a^
Boys (*n*; %)	1050; 52	450; 54%	411; 49%	189; 54%	
Girls (*n*; %)	968; 48	387; 46%	420; 51%	161; 46%	
Age group (*n*; %)					0.160 ^a^
5–6 years old	162; 8	72; 9%	65; 8%	25; 7%	
7–8 years old	713; 35	284; 34%	286; 34%	143; 41%	
9–10 years old	655; 32	275; 33%	286; 34%	94; 27%	
11–12 years old	488; 24	206; 25%	194; 23%	88; 25%	
Age (year; mean ± sd)	8.9 ± 1.8	8.9 ± 1.8	8.9 ± 1.7	8.9 ± 1.9	0.839 ^b^
Weight (kg; mean ± sd)	35.8 ± 11.1	36.4 ± 11.9	35.6 ± 10.4	35.1 ± 10.7	0.123 ^b^
Height (cm; mean ± sd)	137.0 ± 11.7	136.8 ±12.0	136.6 ± 11.3	136.4 ± 12.0	0.900 ^b^
BMI (kg/m^2^; mean ± sd)	18.8 ± 3.4	19.0 ± 3.6	18.7 ± 3.3	18.5 ± 3.0	0.021 ^b^
Urinary iodine (µg/L; median (P25; P75))	129 (88; 181)	116 (79; 164)	137 (99; 194)	138 (96; 189)	0.000 ^c^
Urinary iodine/creatinine (µg/g; median (P25; P75))	126 (83; 183)	114 (78; 165)	132 (87; 192)	142 (93; 205)	0.000 ^c^
Urinary sodium, est. (mg/day; mean ± sd)	2576 ± 782	2587 ± 790	2558 ± 778	2593 ± 770	0.684 ^b^
Salt intake, est. (g/day; mean ± sd) [12]	6.4 ± 2.0	6.5 ± 2.0	6.4 ± 1.9	6.5 ± 1.9	0.684 ^b^

^a^ chi-square; ^b^ ANOVA; ^c^ Kruskal-Wallis. BMI, Body Mass Index; est., estimated.

**Table 2:** Urinary iodine status by sex and age groups.

Variables	UIC (µg/L)				*p* Value	UIC < 100 µg/L		UIC > 300 µg/L (%)		*p* Value	Iodine-to-Creatinine Ratio (µg/g)			*p* Value
	*n*	P25	Median	P75		*n*	%	*n*	%		P25	Median	P75	
Boys	1050	84	134	177	0.002 ^b^	303	29	63	6	0.005 ^c^	89	131	185	0.002 ^b^
Girls	968	91	123	188		331	34	32	3		79	117	182	
5–6 years old	161	110	157	208	<0.001 ^a^	32	20	16	10	<0.001 ^c^	128	189	262	<0.001 ^a^
7–8 years old	711	94	138	192		194	27	42	6		104	149	218	
9–10 years old	654	84	125	174		220	34	26	4		81	121	169	
11-12 years old	487	7	115	160		188	39	11	2		66	89	133	

^a^ Kruskal-Wallis; ^b^ Mann-Whitney; ^c^ chi-square.

**Table 3:** Urinary iodine status and dietary habits.

Food Consumption	UIC (µg/L)					UIC < 100 µg/L		UIC > 300 µg/L		
	*n* (%)	P25	Median	P75	*p* Value	*n*	%	*n*	%	*p* Value
Ilk *^,#^										
<1 glass/day	142 (23)	68	96	151	<0.001 ^a^	72	50	0	0	<0.001 ^c^
1 glass/day	242 (39)	83	120	173		86	36	8	3	
≥2 glasses/day	231 (38)	107	149	207		47	20	19	8	
Yogurt ^#^										
<1 yogurt/day	294 (48)	79	117	171	0.010 ^a^	112	38	10	3	0.018 ^c^
1 yogurt/day	221 (36)	91	129	186		70	32	11	5	
≥2 yogurts/day	100 (16)	102	147	192		8	8	6	6	
Eggs ^#^										
<1 egg/week	181 (29)	88	131	184	0.326 ^b^	53	29	5	3	0.138 ^c^
≥1 egg/week	434 (71)	82	124	181		152	35	22	5	
Fish										
≤1 times/week	413 (21)	88	128	190	0.569 ^b^	118	29	26	6	0.284 ^c^
>1 times/week	1533 (79)	87	129	180		493	32	69	5	
Premade baby cereal										
≤1 times/week	1708 (89)	87	124	180	0.002 ^b^	551	32	69	4	<0.001 ^c^
>1 times/week	215 (11)	101	140	201		51	24	23	11	
Household iodised salt										
Self-reported										
No	1085 (56)	87	129	180	0.646 ^a^	344	32	48	4	0.921 ^c^
Yes	162 (8)	80	129	184		52	32	10	6	
Unknown	677 (35)	92	130	182						
Measured **										
No	130 (83)	74	120	181	0.001 ^b^	46	35	7	5	0.118 ^c^
Yes	26 (17)	137	173	255		3	12	3	12	

* 1 glass or 250 ml of cow milk; ^#^ data collected from respondents to online questionnaire (*n* = 615); ** iodine content of household salt confirmed by inductively coupled plasma-mass spectrometry (ICP-MS); ^a^ Kruskal-Wallis, ^b^ Mann-Whitney, ^c^ Pearson chi-square.

**Table 4:** Logistic regression models for the association between urinary iodine concentration (UIC) <100 µg/L and consumption frequency of different food items.

Variables		Crude ^a^ OR ^b^ (95% CI)	*p* Value	Adjusted ^a^ OR ^b^ (95% CI)	*p* Value
Milk (*n* = 615)	≥2 glasses/day	1.0	<0.001	1.0	<0.001
	1 glass/day	2.16 (1.43–3.27)		2.20 (1.45–3.34)	
	<1 glass/day	4.03 (2.54–6.37)		3.85 (2.42–6.13)	
Yogurt (*n* = 615)	≥2 yogurts/day	1.0	0.019		
	1 yogurt/day	1.55 (0.90–2.68)			
	<1 yogurt/day	2.06 (1.22–3.47)			
Fish (*n* = 1946)	>1 times/week	1.0	0.164		
	≤1 times/week	0.84 (0.67–1.07)			
Eggs (*n* = 615)	≥1 egg/week	1.0	0.169		
	<1 egg/week	0.77 (0.53–1.12)			
Premade baby cereal (*n* = 1923)	≥1 times/month	1.0	0.001		
	<1 times/month	1.54 (1.20–1.99)			

^a^ Crude OR were calculated using univariate weighted logistic regression models. Adjusted OR were calculated using multivariate weighted logistic regression models. Fully adjusted estimates take into account all three variables (sex, age, and milk) in the model (*n* = 615); ^b^ Risk (OR) of urinary iodine concentration below <100 µg/L. 95% CI–95% confidence interval; OR–Odds ratio.

**Table 5:** Milk consumption * by age group.

Age	<1 Glass/Day		1 Glass/Day		≥2 Glasses/Day		Total	*p* Value
	*n*	% per Age Group	*n*	% per Age Group	*n*	% per Age Group		
5–6 years old	4	10%	19	45%	19	45%	42	0.045 ^a^
7–8 years old	54	21%	109	42%	98	38%	261	
9–10 years old	57	31%	60	32%	68	37%	185	
11–12 years old	28	22%	54	42%	46	36%	128	
Total	143		242		231		616	

* 1 glass or 250 mL of cow milk, ^a^ Pearson chi-square.
